# Mobility improvement of patients with peripheral visual field losses using novel see-through digital spectacles

**DOI:** 10.1371/journal.pone.0240509

**Published:** 2020-10-14

**Authors:** Ahmed M. Sayed, Mohamed Abou Shousha, MD Baharul Islam, Taher K. Eleiwa, Rashed Kashem, Mostafa Abdel-Mottaleb, Eyup Ozcan, Mohamed Tolba, Jane C. Cook, Richard K. Parrish

**Affiliations:** 1 Biomedical Engineering Department, Helwan University, Helwan, Egypt; 2 Bascom Palmer Eye Institute, University of Miami, Miami, FL, United States of America; 3 Electrical Engineering and Computer Science, MSOE University, Milwaukee, WI, United States of America; 4 Department of Electrical and Computer Engineering, University of Miami, Miami, FL, United States of America; 5 Biomedical Engineering Department, University of Miami, Miami, FL, United States of America; 6 Department of Computer Science, American University of Malta, BML, Malta; 7 Faculty of Medicine, Department of Ophthalmology, Benha University, Benha, Egypt; 8 Net Eye Medical Center, Gaziantep, Turkey; Purdue University, UNITED STATES

## Abstract

**Purpose:**

To evaluate see-through Augmented Reality Digital spectacles (AR DSpecs) for improving the mobility of patients with peripheral visual field (VF) losses when tested on a walking track.

**Design:**

Prospective Case Series.

**Participants:**

21 patients with peripheral VF defects in both eyes, with the physical ability to walk without assistance.

**Methods:**

We developed the AR DSpecs as a wearable VF aid with an augmented reality platform. Image remapping algorithms produced personalized visual augmentation in real time based on the measured binocular VF with the AR DSpecs calibration mode. We tested the device on a walking track to determine if patients could more accurately identify peripheral objects.

**Main outcome measures:**

We analyzed walking track scores (number of recognized/avoided objects) and eye tracking data (six gaze parameters) to measure changes in the kinematic and eye scanning behaviors while walking, and assessed a possible placebo effect by deactivating the AR DSpecs remapping algorithms in random trials.

**Results:**

Performance, judged by the object detection scores, improved with the AR DSpecs (P<0.001, Wilcoxon rank sum test) with an average improvement rate of 18.81%. Two gaze parameters improved with the activated algorithm (P<0.01, paired t-test), indicating a more directed gaze on the central path with less eye scanning. Determination of the binocular integrated VF with the DSpecs correlated with the integrated standard automated perimetry (R = 0.86, P<0.001), mean sensitivity difference 0.8 ± 2.25 dB (Bland-Altman).

**Conclusions:**

AR DSpecs may improve walking maneuverability of patients with peripheral VF defects by enhancing detection of objects in a testing environment.

## Introduction

Severe peripheral visual field loss (PVFL) causes mobility problems such as motion estimation [1], postural stabilization [2], and gait stride-to-stride variability [3]. Retinitis pigmentosa, choroideremia, advanced glaucoma, and cerebral microvascular events cause decreased awareness of the surrounding [4]. Marked PVFL increases dependence at home and mobility problems including falls [5]. Intermittent eye fixation, stimulated with visual input rather than the continuous voluntary fixation, is required to avoid hitting obstacles and is impaired in patients with PVFL [6]. Lee and coworkers reported that glaucoma patients perform more saccades, although they miss peripheral objects [7]. Lajoie and associates demonstrated that glaucoma patients exhibit an altered gaze pattern compared to normal subjects, and experience more obstacle contacts [8].

A need exists for new technologies that diminish the adverse effects of PVFL on mobility. Head-mounted display (HMD) technology has been commonly used for this purpose [9–14]. HMD devices aiming to reduce central visual impairments produce generalized visual enhancements, not unique for each patient’s visual field defect, and studies have shown their inability to improve patients collisions rates [14–16]. In addition, optics based visual aids cause a perceived patients image jumps, overlap, and reduced resolution [4,17]. These methods have not gained popularity among patients [4,13]. Efforts have been made to develop low vision aids that overcome PVFL [9,10,13,14,18], but no clinical studies have demonstrated functional improvement [11,19,20]. Current aids lack VF defect quantification as a unique visual profile cannot be applied to improve patient specific visual function [14–16].

We reported a virtual reality (VR) based digital spectacles (DSpecs) [21,22] and tested them in a simulated walking environment. Our previous studies demonstrated the concept of expanding the functional peripheral visual field by utilizing a customized visual augmentation method that considers the patients unique PFVL. However, our former VR DSpecs were cumbersome, isolated patients from their surrounding environment and deprived them from employing common compensatory visual scanning behaviors; namely eye and head scanning [22]. In this study, we report testing of an augmented reality (AR) DSpecs that applied digital video processing strategies unique to each patient in real time. Eye movements and gaze data were captured on a walking track and we compared the gaze behavior to assess eye scanning patterns with the AR DSpecs with and without augmentation.

## Materials and methods

**Fig 1:**
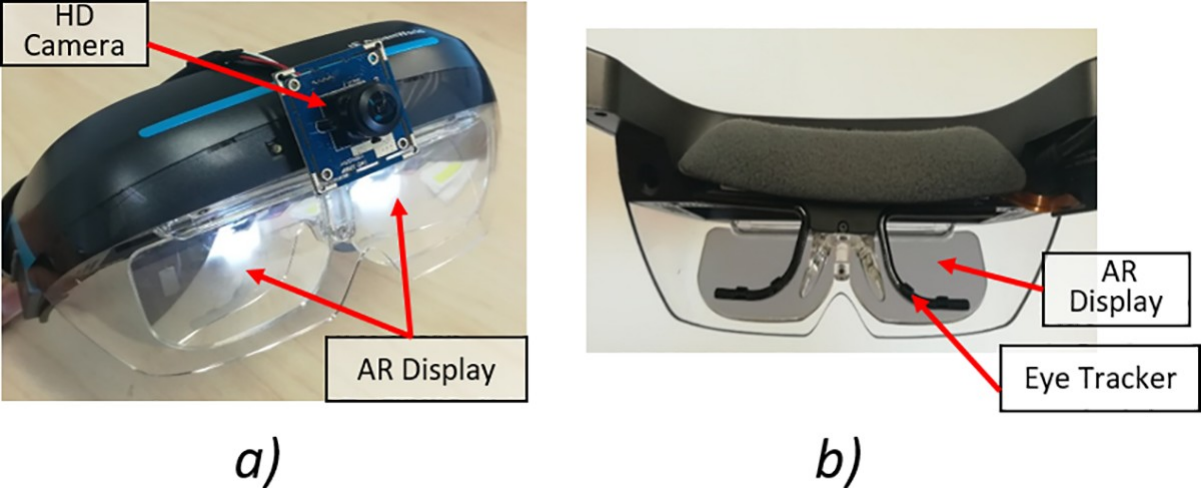
Augmented Reality (AR) Digital Spectacles (DSpecs): a) AR head mounted display (HMD) with a miniature camera. b) The HMD with an integrated eye tracking system.

We utilized a commercial AR HMD (Dreamworld AR, San Mateo, CA, USA) to build the AR DSpecs visual aid (Fig 1A). The AR DSpecs was equipped with a high definition (HD) 2 megapixels, 180^0^ field of view (FOV) miniature camera (Camera sensor: OmniVision, Santa Clara, California, USA) mounted on the central front part of the headset. The camera FOV was digitally limited to 60^0^ horizontally and 40^0^ vertically to match the FOV of the AR headset display. We integrated the visual aid with a wearable eye tracker (Tobii Pro Glasses 2, Tobii Technology, Danderyd, Sweden) that acquired gaze data and wirelessly transmitted it to a control computer (Fig 1B). The AR DSpecs was controlled by a minicomputer (NUC7i7BNK, Intel Core i7-7567U, CPU 3.5GHz, 16GB RAM, Santa Clara, CA) to run the binocular VF testing program, and the microcomputer (STK2MV64CC, Intel Core m5-6Y54, CPU 2.7GHz, 8GB RAM, Santa Clara, CA) to run the video processing algorithm in the walking experiments. All developed algorithms were implemented with C# under Unity (Unity Technologies, San Francisco, California, USA).

### Participants recruitment

The University of Miami institutional review board (IRB) approved the protocol before patient recruitment and we conducted the study in accordance with the Declaration of Helsinki and HIPAA regulations. Participating patients in the study signed a written consent before commencing experimentation. We examined 21 patients recruited from glaucoma and neuro ophthalmology clinics at the University of Miami Miller School of Medicine. All patients had performed either the 30–2 or 24–2 Standard Automated Perimetry (SAP) tests in both eyes, with the Swedish Interactive Threshold Algorithm (SITA) Standard strategy. Three patients were tested with the FASTPAC program testing strategy in both eyes (n = 1), and FAST PAC strategy for one eye (n = 2).

#### Inclusion criteria

1. Availability of Humphrey Zeiss SAP (Carl Zeiss Meditec, California), program 24–2 or 30–2 mean deviation worse than -10 dB in both eyes.
2. Peripheral VF defects in both eyes.
3. Patients with normal mobility who can walk without assistance or a mobility aid.

### AR DSpecs calibration mode: Measuring the binocular visual field

The AR DSpecs binocular VF testing method utilized a modified static SAP technique [23]. We applied a fast thresholding strategy with multi-contrast changing stimuli to test a VF of 60 H X 36 V degrees with 60 stimuli sequences at predetermined positions in a 60 cell grid (6 rows and 10 columns) in each eye. The spatial testing resolution was 6^0^ between each stimulus location and is comparable to SAP testing strategies [23]. We used a background with bright white illumination of 74 lux and presented the stimuli as dark points with different contrast levels in an inverted stimuli pattern (light gray to black stimuli on a white background) in a continuously descending order with digital values between 25 to 0, corresponding to 28 to 0 dB in SAP testing. The stimulus size was 0.563^0^. Patients responded to seeing the stimuli by pressing a wireless clicker. We presented stimuli at randomly selected locations. To confirm patient fixation, the gaze position was continuously monitored with the eye tracking system, and the testing program stopped if the gaze shifted from the center. All 60 responses were arranged in a 6 × 10 matrix and mathematically interpolated with a bicubic function to generate a grayscale VF plot.

For comparison, we constructed binocular integrated visual fields (IVF) by combining the SAP VFs and compared these to the corresponding AR DSpecs binocular VF tests obtained in the calibration mode. We used the maximum sensitivity integration model described by Crabb and coworkers to construct the reference IVFs [24]. We calculated the VF mean sensitivity value for each test type and compared them with Bland-Altman analysis and Spearman Rank-Order correlation. Comparisons and correlation calculations were performed at the common central area in the two measurement methods with either the 24^0^ or 30^0^ test. We did not design the AR DSpecs VF testing strategy to mimic the results of commercially available SAP testing equipment, but to identify the size and relative location of defects and develop image remapping algorithms.

### Image remapping algorithm

Image manipulations were applied to fit the captured video images of the unseen VF into the remaining intact VF. Mathematical geometric calculations were performed to apply image remapping operations of rescaling and shifting. The intact VF identified with the VF calibration mode was fed to the image remapping algorithm to estimate an image rescaling and shifting strategies relative to the original image size. The program applied the two remapping parameters on the captured video images from the front camera to expand the FOV in real time. Our recent studies [21,22] provide detailed descriptions of the remapping algorithms.

### Hand coordination test

We investigated the possibility that the AR DSpecs image manipulation algorithms could worsen hand-eye coordination by testing coordination with and without the augmentation algorithms. Patients were instructed to grasp three different sized objects: a green pen, an empty white/brown coffee cup, and a white/orange palm-sized ball at a distance of 60, 75, and 90 cm. The grasping order of each object was randomly assigned. The patients performed the hand coordination test first without the AR DSpecs to quantitate normal hand coordination capabilities, and then repeated it while activating the video remapping algorithms. We recorded the number of trials to achieve successful grasping in both conditions.

### Walking track description

We built a walking track to determine if using the AR DSpecs could improve the ability of patients with PVFL to detect peripheral objects and safely navigate in an environment that mimicked daily mobility activities. The track was adapted from a study reported by Lajoie and associates to measure differences in mobility between normal and glaucoma patients [8].

**Fig 2:**
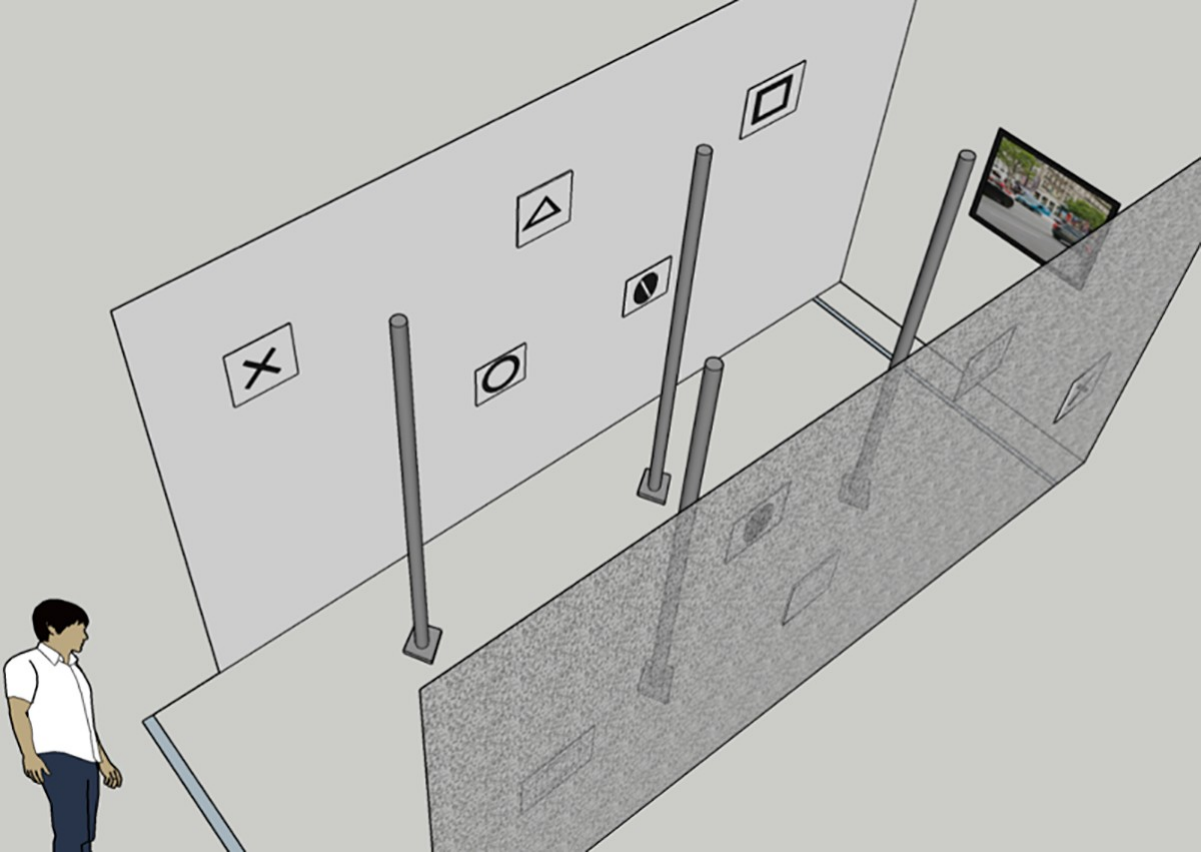
Experimental set-up: 3D view of the walking track.

We tested patients on a walking track, 15 feet long and 5 feet wide (Fig 2). The track included four long poles as vertical obstacles. Ten peripheral shapes were positioned on the track walls at two levels; five shapes above the horizontal line of sight at 6.5 ft., and five shapes placed located below the horizontal line of sight at 3.5 ft. These included five shapes: circle, triangle, square, stop sign, and the letter ‘X’. This display required the patient to simultaneously multitask by avoiding obstacles while identifying shapes, thereby simulating a real-life activity. We positioned a video monitor that showed a dynamic street scene to produce a realistic environment at the end of the walking track. Patients were instructed to look at the central monitor to decrease the eye scanning compensation mechanism of their VF defects on the experiment outcomes. We modified the test by changing locations of obstacles and shapes. Patients walked twice at normal speed through the track with the AR DSpecs image remapping activated and two additional times without the AR DSpecs, uncorrected vision (n = 17) and with prescription glasses (n = 4). The trial sequence was randomly generated. We calculated the test scores as the average of the two walking trials for each test condition. The patient also walked twice without activating the AR DSpecs remapping algorithms. The images from the front camera were displayed directly to the AR DSpecs without modifications to test the possible placebo effect of the device. We did not tell the patient which profile was used. The patient walked with different obstacle/shape arrangements each time, to minimize a learning/memory effect.

A study team member closely observed all patients and walked behind them during the test. Patients wore a safety gait belt for support to minimize the likelihood of injury. An opaque screen occluded vision at the track starting point before commencing each trial to reduce the effect of spatial memory effect and initial scanning efforts that could occur by memorizing the obstacle/shape locations from the previous trial. Patients walked without AR DSpecs through the track and exercised the test responses (2 to 5 rounds, average: 3.5), to confirm they understood the test before beginning the study.

### Mobility and gaze scores

Video recordings of the walking trials were utilized to determine the patients obstacle crossing or touching events, while audio recordings were used to score the patients verbal shape recognition responses. Test efficiency was judged by two sets of quantitative measures: mobility and gaze scores. Mobility and kinematic measures included track completion time, obstacle avoidance events, and the number of detected peripheral shapes. One observer documented the patient maneuvering and shape recognition scores. Later a different observer validated the scores by comparing the patient response with the audio and video recordings. The high-speed eye tracker recorded gaze data to determine if patients shifted gaze to avoid obstacles and identify objects. Gaze fixations were defined as stable gaze positions for a minimum period of 60 milliseconds. Gaze shifts were detected from the raw eye tracking data with a velocity-threshold identification classification algorithm. We defined a velocity threshold of 30^0^/second and values below that were classified as fixations, and higher values were categorized as saccades [25].

The eye tracking system recorded the two trial conditions when the patient walked with AR DSpecs; with and without image remapping (two conditions of gaze data). Based on similar studies [8,26], we used the following eye gaze scores:

1. Number of gaze fixations/second—the frequency of search or scanning attempts.
2. Variance of the fixation locations–geographic dispersion of the fixation locations.
3. Mean fixation period (milliseconds)–time to see and recognize an object.
4. Gaze location score—percentage of gaze vectors directed toward the center of the VF. Higher scores were given to gaze directions heading towards the center. We used a score range from 4 to 1 with decrements of 1. Gaze fixations located at the central FOV of 10^0^ diameter were given a score of 4. Fixations in the area between the central 10^0^ to 20^0^ diameter were scored 3, between 20^0^ and 30^0^ FOV were scored 2, and the lowest score of 1 was given to fixations located away from the 30^0^ central FOV.
5. Spatial temporal gaze direction score—fixation durations divided by the walking time per track multiplied by the gaze direction score (parameter number 4). Larger average values of the spatial temporal gaze direction indicated that the gaze is allocated farther ahead for a greater amount of time than focused on nearby objects.
6. Mean saccadic amplitude (degrees)—mean distance between subsequent fixations. This score reflects the average range of the visual scanning pattern.

Gaze directed toward the central area indicated a route planning pattern that reflected greater patient peripheral awareness and a decreased need for extensive scanning during walking. Eye tracking parameters and scoring were calculated with a custom MATLAB script (MATLAB R2019b, MathWorks, Inc., Natick, MA, USA), after exporting the pre-processed eye tracking data with the Tobii Pro Lab software (Tobii Technology, Danderyd, Sweden).

We evaluated the recorded eye tracking data by determining validity of the acquired gaze samples. The eye tracking system calculates a gaze data validity measure as a percentage of the recording session duration, by considering the amount of data losses and random variations in the gaze direction signals. We set a data validity measures > 35% as a threshold to ensure that the recording had sufficient valid samples for statistical interpretation. Based on this criteria, 17 patient trials recordings were included and 4 were excluded.

### Statistical analysis

Statistical analysis determined the difference level between two conditions; walking performance with the unaided vision and with the DSpecs activated image remapping. The gaze parameters (fixation rate, variance, period, gaze location and temporal-directional scores, and saccadic amplitude) were analyzed to determine the difference level between two additional conditions: gaze behavior with and without image remapping algorithms. Significance and descriptive statistics were computed using SPSS (IBM Corporation, Armonk, NY). Means ± standard deviations (SD) described binocular VF measurements errors. Wilcoxon rank-sum tests were used to test for significance between the patients walking scores, with and without the AR DSpecs, and the placebo AR DSpecs effect. We used Spearman Rank-Order to assess the correlation of VF defect characterization measures, mean deviation (MD), pattern standard deviation (PSD), and the Visual Field Index (VFI) and the walking track average scores [27,28]. For each metric we used the values from the eye with the least visual field loss in the analysis. We used paired t-tests to determine the mean differences in gaze parameters between the with and without image remapping two walking conditions.

## Results

### AR DSpecs calibration mode: Binocular VF measurements

**Fig 3:**
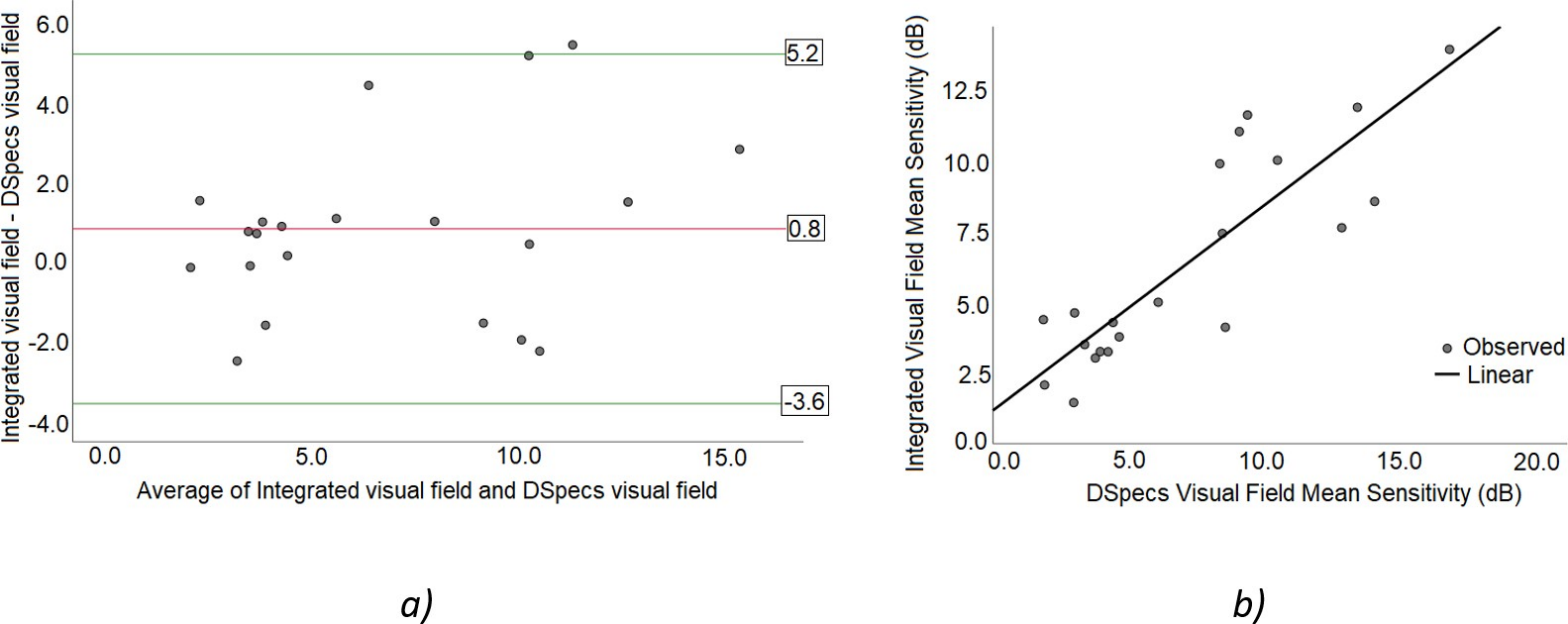
Assessment of AR DSpecs binocular visual field (VF) measurements for 21 patients: a) Bland-Altman analysis of the mean sensitivity values for AR DSpecs and Integrated VF tests (difference: 0.8 ± 2.25 dB). b) Mean sensitivity linear correlation between the two VF testing methods (R = 0.86, P<0.001).

**Fig 4:**
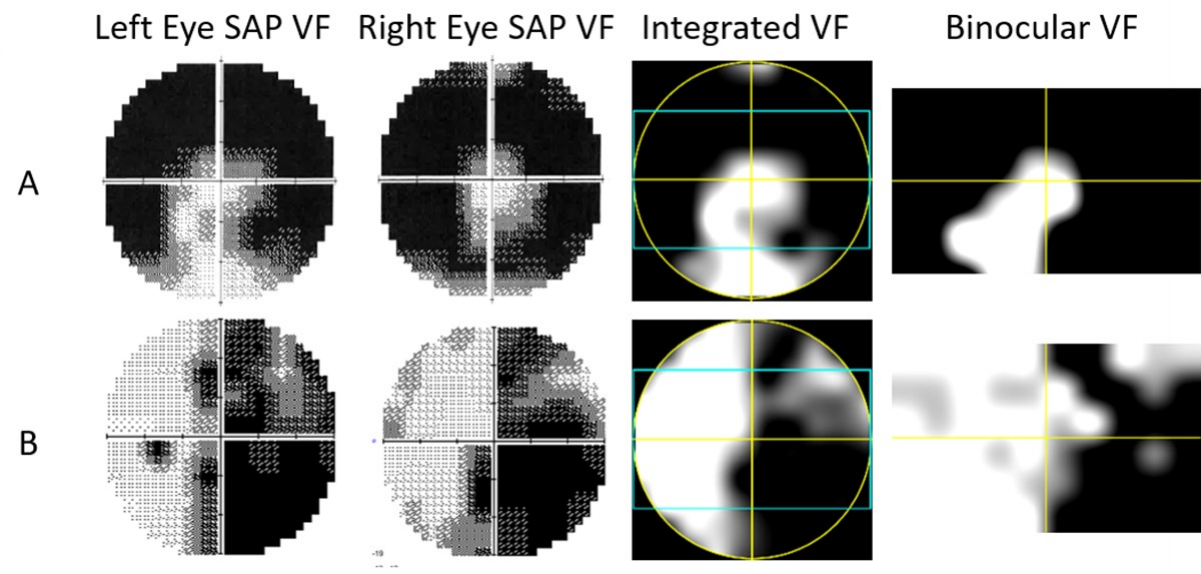
Binocular visual field (VF) measurements for two patients. A) Retinitis pigmentosa patient. B) Stroke patient. Both patients were tested with 30–2 monocular Humphrey SAP. First column: left eyes monocular SAP. Second column: Right eyes Monocular SAP. Third column: binocular Integrated VF (IVF) constructed by merging the two monocular fields based on the maximum sensitivity model. Fourth column: AR DSpecs binocular VF measurements. The blue rectangles in the third column represent measurement area of the AR DSpecs for comparison purposes.

Patients ages ranged from 28 to 80 years with a mean of 55.5 ± 13. Spherical equivalent ranged from 0.375 to -3.5 Diopters. The mean sensitivity values of the AR DSpecs IVF tests versus reference IVF measurements were 0.8 ± 2.25 dB (Bland-Altman analysis, mean difference ± SD). Of the measurement points, 4.5% were located outside the limit of agreement region (5.2 dB to -3.6 dB, 95% confidence interval, Fig 3A). Additionally, we found positive correlation between the two methods, with a correlation coefficient of R = 0.86 (P< 0.001, Fig 3B). Two examples demonstrate the comparability of the reference binocular IVF and the AR DSpecs measured binocular IVF. The right eye VF of a patient with retinitis pigmentosa demonstrates tunnel vision and the left eye exhibits an intact region in the inferior hemifield.

(Fig 4A) Combining both VFs resulted in the IVF that is similar to the left eye VF and includes intact areas that compensated for the right eye defects. (Fig 4A, 3^rd^ column) The AR DSpecs binocular IVF is shown for comparison. (Fig 4, 4^th^ Column) A VF, associated with cerebral microvascular events, in the left hemifield of both eyes shows minor defects, and the right hemifield documents more extensive damage in the inferior region of the hemifield. (Fig 4B) The AR DSpecs binocular IVF generates a common defect in the inferior-right quadrant, and less severe defects in the superior-right quadrant.

### Hand coordination test

All patients successfully grasped 3 objects from the first trial with unaided vision. With the AR DSpecs, 16 of 21 patients (76.2%) grasped the 3 objects on the first trial. Four grasped two objects in the first trial and one object in the second trial, and the fifth grasped one object from the first trial and two objects in the second trial. These five patients repeated the test twice until they successfully grasped the three objects.

### Walking track scores

**Fig 5:**
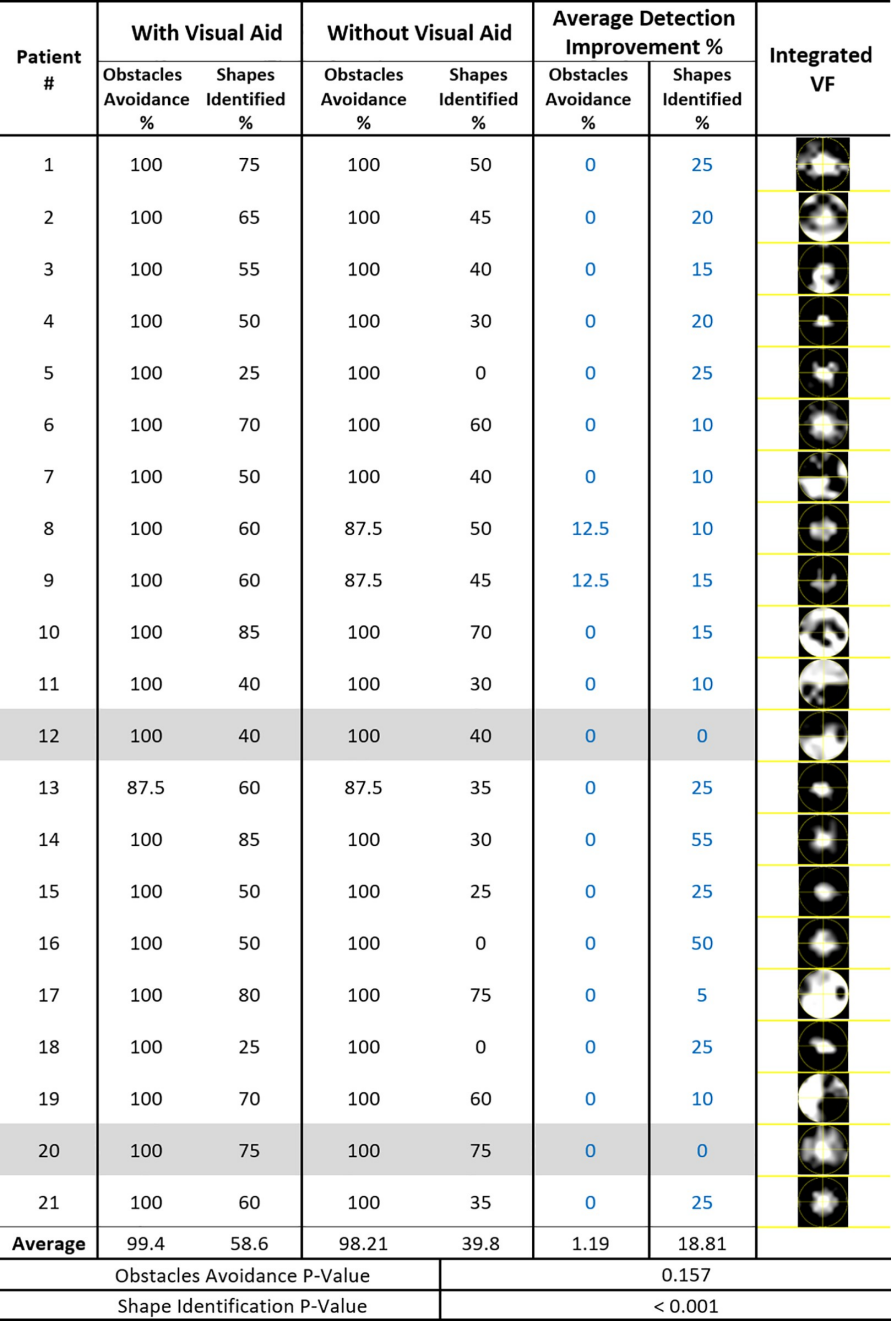
Walking track test scores for 21 patients with and without the digital spectacles.

We calculated mobility scores as the percentage of successful obstacle avoidances (Fig 5: columns 2 and 4, with and without AR DSpecs, respectively) and correct shapes identification responses (Fig 5: columns 3 and 5, with and without AR DSpecs, respectively). Each task score improvement was calculated as the difference between the corresponding scores: after and before walking with the AR DSpecs (Fig 5: columns 6 and 7, obstacle avoidance and shape identification improvement scores, respectively). The average score improved 1.19% in the obstacle avoidance task (P = 0.16), and 18.81% for shape identification (P<0.001, Wilcoxon rank sum test). The AR DSpecs improved the shape identification score in 90.5% of subjects (19 out of 21 patients, column 7 of Fig 5) but two patients showed no improvement in peripheral detection; patients 12 and 20, as highlighted in (Fig 5). Track completion times for the two walking conditions were 15.5 ± 11 seconds and 17.3 ± 9.4 seconds (P = 0.14).

**Fig 6:**
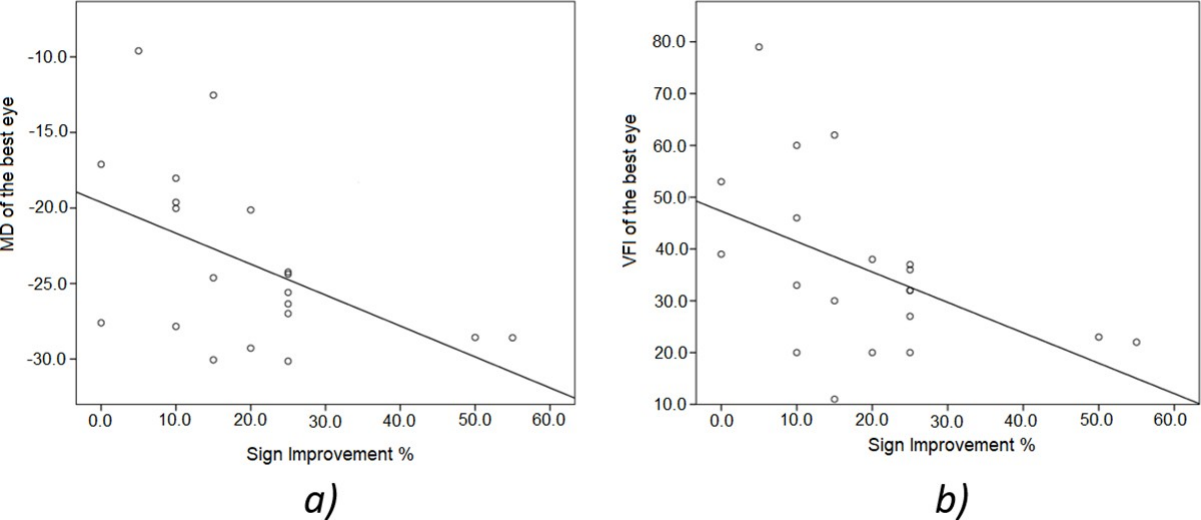
Correlation analysis: a) Mean deviation (MD) linear correlation with percentage of shape improvements with AR DSpecs (R = -0.53, P = 0.02). b) Visual field index (VFI) linear correlation with percentage of shape improvements with AR DSpecs (R = -0.54; P = 0.02).

We correlated walking mobility scores with three standard SAP parameters: mean deviation (MD), visual field index (VFI) and pattern standard deviation (PSD) in the eye with the least VF loss. The AR DSpecs walking improvement scores were correlated with MD and VFI (R = -0.53; P = 0.02 and R = -0.54; P = 0.02, respectively, Spearman Rank-Order; Fig 6). The PSD was not correlated with AR DSpecs improvements (R = -0.25; P = 0.29). Age was not correlated with the scores (R = -0.29; P = 0.21).

The placebo effect test of the visual aid was performed and comparisons between the patient responses in each condition are listed (Table 1). With similar analysis in the AR DSpecs testing, the differences between the walking scores in the two conditions were not significant (Wilcoxon rank sum tests, P = 0.71 and 0.57, respectively). Track completion times were 15.53 ± 11.05 seconds and 17.32 ± 14.87 seconds, respectively, (P = 0.23*)*.

**Table 1:** Testing placebo effect: Walking track test scores for 21 patients with and without the digital spectacles, image remapping was not activated in the spectacles.

Patient #	With Placebo Visual Aid		Without Visual Aid		Average Detection Improvement %	
	Obstacles Avoidance %	Shapes Identified %	Obstacles Avoidance %	Shapes Identified %	Obstacles Avoidance %	Shapes Identified %
1	100	70	100	50	0	20
2	100	55	100	45	0	10
3	100	35	100	40	0	-5
4	87.5	30	100	30	-12.5	0
5	100	10	100	0	0	10
6	100	55	100	60	0	-5
7	100	45	100	40	0	5
8	100	55	87.5	50	12.5	5
9	50	0	87.5	45	-37.5	-45
10	100	80	100	70	0	10
11	100	20	100	30	0	-10
12	100	35	100	40	0	-5
13	100	35	87.5	35	12.5	0
14	100	30	100	30	0	0
15	100	25	100	25	0	0
16	100	0	100	0	0	0
17	100	75	100	75	0	0
18	100	10	100	0	0	10
19	100	45	100	60	0	-15
20	100	70	100	75	0	-5
21	100	55	100	35	0	20
**Average**	97.02	39.76	98.21	39.8	- 1.19	0
Obstacles Avoidance P-Value				0.71		
Shape Identification P-Value				0.57		

### Eye tracking scores analysis

**Fig 7:**
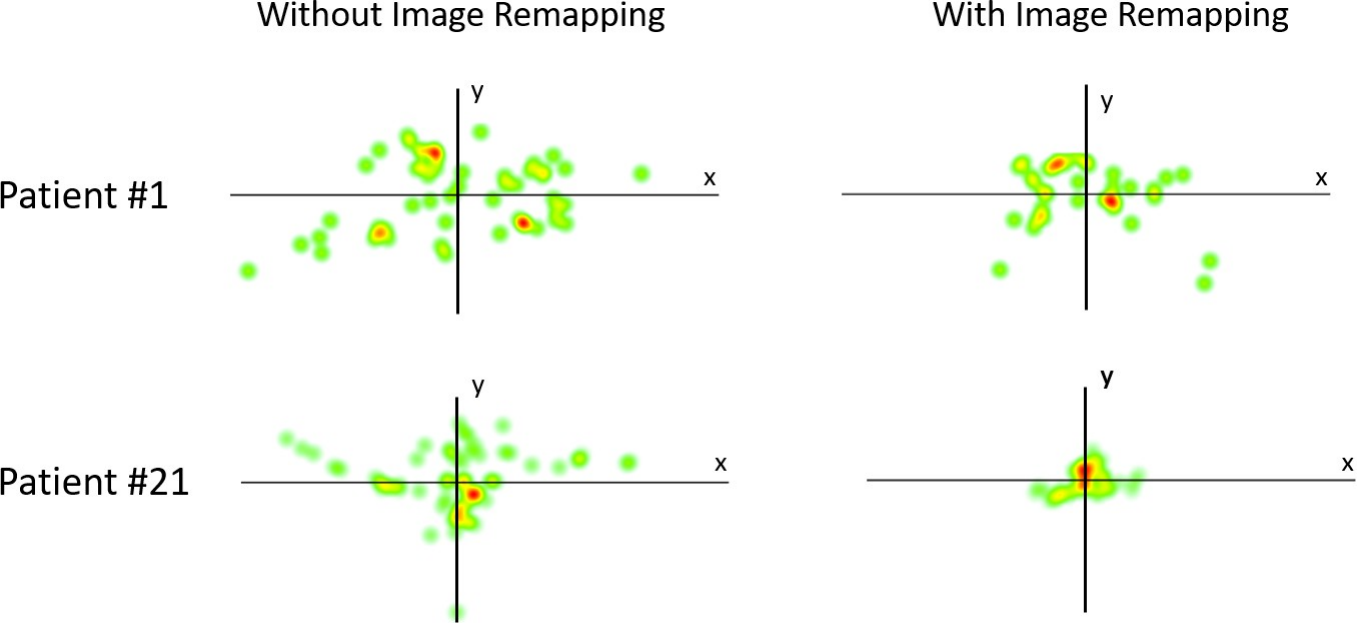
Illustration of eye scanning behavior for two patients with gaze heat maps (Fixation locations plot). Left column shows a more scattered gaze as the patients walked with the visual aid without activating image remapping. Right column shows a more centric scanning behavior after activating image remapping.

Two examples of the acquired gaze data after averaging the two walking trials recordings for each condition are shown (Fig 7). Gaze heat maps representing location and duration of eye fixations were plotted to demonstrate the dispersion of gaze fixations while walking. Gaze fixations were more widely distributed over the AR DSpecs display area when patients walked without activating video remapping (condition 1, patients walking without visual compensation). With the activated video remapping algorithm (condition 2, patients walking with visual compensation), the fixations were more focused on the central FOV, indicating that they did not perform comparable eye scanning to detect peripheral objects while walking. (Right Column Fig 7).

With the custom scoring MATLAB script, we calculated gaze data measures and scores to quantitatively characterize the eye movement behavior and determine statistical significance. Mean and standard deviation values of the six metrics were used to describe the 17 pair of gaze data. (Table 2) Fixation rates were comparable in both conditions, with an average frequency of 2.3 fixations per second. No statistical difference between the two conditions regarding the search attempts was noted (P = 0.686, paired t-test). Variance of the fixation locations in the first condition was about twice the variance in the second condition, with a significant statistical difference (P = 0.004). In the uncompensated condition, patients had a greater proportion of more widely distributed fixations than in the compensated group. A slightly decreased mean fixation period (about 8 milliseconds difference) in the second condition was noted, but was not significantly different than in the first condition (P = 0.394). Central fixation scores were different between the two conditions (P = 0.002), with higher scores in the second condition, indicating that patients were looking more toward the central FOV and end of the track, while seeing peripheral shapes with their expanded peripheral vision. The spatial temporal score was slightly higher in the second condition, indicating a gaze directed farther ahead for a greater amount of time, but this was not significant (P = 0.065). The mean saccadic amplitude between fixations in the second condition was less than the first with about 1.1^0^, reflecting a smaller average range of scanning, although not statistically significant (P = 0.541).

**Table 2:** Gaze average descriptive parameters and statistical analysis with the DSpecs under two conditions: With and without image remapping.

Descriptive Statistics	Fixation Rate (Fix/sec)	Variance of Fixation Locations (Degrees)	Mean Fixation period (mSec)	Central Fixations score	Spatial Temporal Score	Mean Saccadic Amp (Degrees)
**DSpecs without Remapping**	2.26 ± 1.05	40.53 ± 29.29	190.96 ± 67.99	3.67 ± 0.19	32.55 ± 19.85	5.26 ± 2.33
**DSpecs with Remapping**	2.34 ± 1.07	22.20 ± 16.59	182.63 ± 62.35	3.81 ± 0.14	34.47 ± 21.23	4.21 ± 2.1
**Significance of paired t-tests**						
**(P-Value)**	0.686	0.004	0.394	0.002	0.065	0.541

## Discussion

We designed, implemented, and tested a new AR based DSpecs that applies video processing to a captured scene, and accordingly produce a unique augmentation profile specifically generated for each patient. Average detection improvement percentage of peripheral objects was substantially more, 18% with the AR DSpecs, with significantly less eye scanning attempts. We believe this is the initial report to demonstrate the use of a digital visual aid with a customizable visual field expansion algorithm to enhance mobility and eye scanning patterns of patients with PVFL on a walking track. We determined that the remapping and augmentation profiles can affect the way patients scans and searches the environment, as the patients fixated further ahead and were more focused around the central FOV. However, we found no other condition differences in the gaze scores with the use of the device. The effect of eye scanning as a compensatory mechanism is important, as patients use this to compensate for VF defects in different types of activities [26,29–32]. In the walking track, lower central fixation scores without the remapping condition indicate that the patient gaze was shifted more toward nearby objects, while higher scores with the activated AR DSpecs suggested that patients were looking farther ahead. This observation was in agreement with a previous study that compared normal subjects and glaucoma patients gaze behavior in a walking test [8]. This implies that with AR DSpecs, our patients were demonstrating more normal eye movement behavior.

Mobility testing has been reported in PVFL patients [8,33,34], to assess the value of new technologies to help patients avoid collisions and assess their perceived FOV. Some implementations with AR headsets minified scene images in front of the eye, including the study by Trese and coworkers [9], however, no mobility tests were performed as we did with our device. Peli and associates [11,20] used an HMD to display expanded contours of peripheral objects delineating their boundaries and successfully increased the usable FOV for patients with retinitis pigmentosa, although it did not improve patients mobility performance in a virtual obstacle course [19]. Our study suggested that the AR DSpecs might be of value to these patients. Wittich and coworkers assessed a commercial visual aid, where only central capabilities showed improvement, and mobility was not improved in a questionnaire based study [16].

Our see-through open AR prototype allowed the patients to use their residual intact peripheral vision while wearing the AR DSpecs, unlike our former VR DSpecs [21,22], that blocked the entire FOV. Two of our patients had central ring scotomas with a “doughnut” VF defect pattern (Fig 5: patients 2 and 10), and both benefited from the AR DSpecs (20%, 15% peripheral identification score, respectively). They likely utilized their mid periphery through the semi-transparent glass of the AR DSpecs, while the central intact area benefitted from the projected remapped image. Hand coordination tests also confirmed that a good level of motor and visual coordination can be maintained with its use, although some patients required training to achieve this effect.

Our patients performed better with the AR DSpecs in the peripheral shape recognition task, while in the obstacle avoidance task they performed in a manner comparable to their natural vision. The walking test positioned the obstacle location at the central 20−30^0^ diameter FOV that was an area visible to patients with unaided vision. This was not the case with the peripheral shapes located in the 50−60^0^ diameter FOV. The AR DSpecs could acquire videos covering this wider FOV and fit them in the intact VF. The AR DSpecs may have improved the peripheral awareness without adversely affecting central vision or coordination as seen in the obstacles track scores and hand coordination tests, which are positive characteristics of our visual aid unlike that of other devices [14–16].

Nineteen patients scored higher with the use of the AR DSpecs, with some participants having a substantial improvement. For example, patients 14 and 16 (retinitis pigmentosa) scored higher by detecting more peripheral shapes (55 and 50%, respectively). We believe that two factors may have affected the AR DSpecs performance in augmenting the patients FOV. First, the VF defect severity, as demonstrated by the correlation analysis of the MD and VFI metrics, the more severe the defect, the more likely the AR DSpecs would be beneficial. Second, the effectiveness of the AR DSpecs video remapping operations and the range of measured VF sensitives, could affect the augmentation performance. The gaze scores demonstrated that the patients utilized the AR DSpecs augmentation profile while walking, as they were looking more toward the central FOV. They also performed fewer scanning attempts with the AR DSpecs while walking. Excessive eye scanning attempts in the non-augmented walking condition compared to the augmented condition, likely compensated for VF losses during the test [26,29–32].

Our study has several limitations. We did not record nor measure the head scanning movement effects. We attempted to limit head movements by encouraging fixation toward a monitor located at the end of the walking track, but could not completely eliminate this artifact. However, as eye movements are faster than head movements, they are the main scanning mechanism [32]. Although head scanning is an important compensatory factor, it would have a minor effect on the walking performance. Another limitation is the type of obstacles we used in the walking track. Obstacle avoidance scores differences were not different in the two testing conditions, as the obstacles were close to the central FOV and were fixed in location, so it is possible that patients could identify and avoid most of the obstacles. A faster and changing testing environment could be used to demonstrate if the AR DSpecs are beneficial in an everyday activity.

We will improve the AR DSpecs technology and study this application in more realistic dynamic testing environments. We believe that the AR DSpecs greatest potential benefit would be helping patients to detect moving peripheral objects in the periphery without scanning. The current AR prototype has a 60^0^ horizontal 36^0^ vertical FOV that was adequate to demonstrate the possible efficacy of expanding the peripheral VF. However, a more practical FOV for daily life activities would approximate 100^0^ horizontal, comparable to the field covered by prescription glasses. We anticipate designing a more elaborate video remapping algorithm to take into consideration different types and patterns of VF defects, as well as a larger dynamic range of VF measurement sensitivities. Future directions to improve the vision augmentation algorithm include nonlinear operations, such as fisheye transformations, to facilitate fitting even more of the peripheral FOV into the remaining intact VF.

## Supporting information

S1 Table Dataset of results for the recruited 21 patients. **Table 1.** Walking scores and improvement ratios with the augmented reality digital spectacles (AR DSpecs). Integrated visual field test along with test paramters for bothe eyes. **Table 2.** Eye tracking data for the two conditions: with and without image remapping. **Table 3.** Mean visual field measurements using AR DSpecs and standard automated perimetery.
(XLSX)
